# Can Academic Achievement in Primary School Students Be Improved Through Teacher Training on Emotional Intelligence as a Key Academic Competency?

**DOI:** 10.3389/fpsyg.2019.02976

**Published:** 2020-01-10

**Authors:** Teresa Pozo-Rico, Ivan Sandoval

**Affiliations:** ^1^Department of Developmental Psychology and Didactics, University of Alicante, Alicante, Spain; ^2^Departamento de Formación Básica, Escuela Politécnica Nacional, Quito, Ecuador

**Keywords:** primary school, academic achievement, emotional intelligence, teacher continued training, methodology and didactics, satisfaction

## Abstract

**Background:**

The primary aim of the current study was to develop a multi-methodological teacher training program based on emotional intelligence (EI) as a key competency in order to improve student academic achievement based on two methods: face-to-face instruction and game-based e-learning instruction.

**Methods:**

Seventy-four primary education teachers and their 2069 students were randomly assigned to three groups. The first group of teachers (*n* = 23) were trained to use a face-to-face method. The objective of the training was that the teachers would be able to implement EI into their teaching to improve academic achievement in their students (*n* = 645) using face-to-face instruction. For the second group (*n* = 28), the teachers were trained to use an e-learning gamification method. Similar to the first group, the objective of the training was that the teachers would be able to implement EI into their teaching to improve academic achievement in their students (*n* = 758) using e-learning gamification instruction. The third group of teachers (*n* = 23) served as the controls and did not receive any special training, nor did they implement EI into their teaching (*n* = 666).

**Results:**

Implementation of EI into classroom teaching effectively improved academic achievement in primary school students using both methods. However, there was a greater increase in academic achievement and higher teacher satisfaction in the game-based e-learning group. No significant differences in student achievement were observed in the control group.

**Conclusion:**

Emotional intelligence as a key academic competency.

## Introduction

### Theoretical Framework

Emotional intelligence (EI) was first defined by Mayer and Salovey (1993) as a “type of social intelligence that involves the ability to monitor one’s own and other’s emotions, to discriminate among them, and to use the information to guide one’s thinking and actions” (p. 433). Since then, numerous studies have been carried out to conceptualize the topic, develop an EI measure, and explore EI as a key competency in education and training to improve performance in different areas (Schutte et al., 1998; Petrides and Furnham, 2000; Mayer et al., 2001; Caruso et al., 2002; Hall et al., 2019).

The use of EI as a key competency to improve academic achievement in primary education has been the subject of many studies (Pangiras et al., 2012; Temple-Harvey and Vannest, 2012; Pozo et al., 2014; Morente et al., 2017; Gilar-Corbi et al., 2018; Howard and Cogswell, 2018; Petrides et al., 2018). However, it is necessary to investigate applied programs with effective methodologies in order to train teachers to serve as enterprising leaders of EI educational projects in the classroom.

In addition, emerging evidence indicates there may be an association between EI as a key competency and school performance (Viguer et al., 2017; Petrides et al., 2018; Wolf et al., 2018) and as a competency with a mediating effect on well-being (Pangiras et al., 2012), happiness (Karavasilis et al., 2010; O’Connor, 2012), positive relationships, and a democratic and tolerant environment in the classroom (Morente et al., 2017; Ferres et al., 2018; Macias Garcia et al., 2018).

In the same way, solid evidence in the scientific literature link competencies with a behavioral approach to emotional, social, and cognitive intelligence (Boyatzis, 2008). Additionally, several studies link competencies with a behavioral approach to emotional EI and as behavioral manifestations of talent (Boyatzis, 2006, 2009). In fact, this robust perspective of the EI behavioral approach provides a more holistic theory of EI because it explains how recognizing, understanding, and using emotional information leads to effective or superior performance (Boyatzis, 2018). So, the findings of previous studies raises three key research questions:

Research Question 1: Does academic performance measured by student school records improve after the 7-week teacher training program on EI as a key competency in primary school education?

Research Question 2: Is there a difference in the change in academic performance between face-to-face instruction and game-based e-learning instruction?

Research Question 3: How does the 7-week teacher training program affect teacher satisfaction?

This study’s hypotheses were as follows: (1) academic performance improves (according to student school records) after the 7-week teacher training program, (2) both of the proposed methods (face-to-face instruction and game-based e-learning instruction) improve academic performance in primary school students, and (3) teachers are satisfied with their participation in the 7-week teacher training program.

### The Content, Methods, and Characteristics of the 7-Week Teacher Training Program

The 7-week teacher training program was designed to provide multi-methodological training to primary school teachers on implementing EI into their teaching as a key competency to improve academic achievement in their students. As explained above, there were two training modalities:

(1) Implementation of EI into teaching as a key competency to improve academic achievement through face-to-face instruction.
(2) Implementation of EI into teaching as a key competency to improve academic achievement through game-based e-learning instruction.

Although the methods were different, the objectives and contents of the programs were the same. The two programs were both delivered in seven sessions (one 5-h session per week, for a total of 7 weeks). The design of the program was based on Mayer and Salovey’s four-branch model (1997): (1) understanding emotions; (2) identifying emotions; (3) expressing and using emotions; and (4) managing emotions. The session topics were as follows: (1) EI as a key competency in primary school, (2) understanding emotions, (3) identifying emotions, (4) expressing emotions, (5) using emotions, (6) managing emotions, and (7) conclusions.

After each session, an education project was completed in the classroom with the primary school students in order to transfer the knowledge on EI from teacher to student and capacity building was focused on to improve academic achievement. In addition, application of the two different methods were as follows:

• First session: *EI key concepts* were taught in order to improve academic achievement in primary school students through face-to-face lessons in the first method and a virtual learning environment in the second method.
• Second session: *Understanding emotions* (and also academic self-esteem, self-realization, and emotional self-consciousness) was taught in order to improve academic achievement in primary school students through roleplay illustrating the importance of understanding emotions. A final theater performance on understanding emotions was conducted for the families of students in the face-to-face group, and an understanding emotions video game created by the students for playing with their families was used for the game-based e-learning group.
• Third session: *Identifying one’s own emotions and the emotions of others* (along with intrapersonal and interpersonal relationships, empathy, and social responsibility) was taught to the primary school students. In the face-to-face method, the students participated in an assembly in which they drew pictures of their classmates with different facial expressions that reflected different emotions and played a game that involved identifying emotions. In the game-based e-learning method, the activity involved virtual painting of classmates.
• Fourth session: Throughout the academic year, *expressing emotions* was taught to the students by having them use self-drawn pictures of their own facial expressions to communicate with the teacher or with the other students about their feelings, especially in conflict situations, in dealing with anxiety before exams, or for frustration over grades. A weekly assembly was used for the face-to-face group, and a weekly e-learning discussion forum was used in the game-based e-learning group.
• Fifth session: *Using the power of positive emotions* in order to improve one’s own positive feelings and the positive feelings of others and *using emotions to solve problems* was taught in order to improve academic achievement in the students through the emotional roadmap mural painting in the face-to-face method and a virtual mural painting upload in a learning environment in the game-based e-learning method.
• Sixth session: *EI strategies and their effectiveness* were taught to the teachers in order to improve academic achievement in the students through theory and group discussion on the analysis and solution of real EI difficulties in class. The students participated in activities that involved positive reappraisal through roleplay and drills in which families participated. A video recording was created and delivered to families by students in the face-to-face group or uploaded and made available online in the game-based e-learning group.
• Seventh session: A *summary of the program, questions, and conclusions* were discussed with the students through a weekly assembly in the face-to-face method and a weekly e-learning discussion forum in the game-based e-learning method.

Finally, for further clarification, the content, methods, and characteristics of the 7-week teacher training program have been summarized (see Table 1).

**TABLE 1:** Content, methods, and characteristics of the 7-week teacher training program.

**Lesson**	**Goal**	**Face-to-face**	**Game-based e-learning**
1	Emotional intelligence (EI) key concepts	Traditional lesson	Virtual learning environment
2	Understanding emotions	Illustration of role play	Theater performance
3	Identifying emotions	Assembly for drawing pictures of classmates	Virtual paintings of classmates
4	Expressing emotions	Assembly regarding self-drawn emotion pictures	E-learning discussion forum about self-drawn emotion pictures
5	Positive emotions	Roadmap mural painting	Mural painting upload in a learning environment
6	EI strategies	Positive reappraisal through role play and drills	Positive reappraisal through a video recording
7	Summary	Conclusions reached through an assembly	Conclusions reached through an e-learning discussion forum

## Materials and Methods

### Participants

A total of 74 primary school teachers (with their 2069 students) participated in this study. They participated voluntarily in this research study and were randomly assigned to three experimental conditions. The first group (*n* = 23) participated in the teacher training program with the objective of implementing EI into their teaching to improve academic achievement in their students (*n* = 645) through face-to-face instruction. The second group (*n* = 28) participated in the teacher training program with the objective of implementing EI into their teaching to improve academic achievement in their students (*n* = 758) through game-based e-learning instruction. The third group (*n* = 23) served as the control group and did not receive an special training, nor did the members of the group implement EI into their teaching (666 students). Of the entire sample of 74 teachers, 48.6% were women and 51.4% were men, and the average age of the teachers was 42.5 years, with a standard deviation of 1.48. There were 2069 students, 51.1% were girls and 48.9% were boys. The average age of the students was 8.48 years, with a standard deviation of 1.49.

### Instruments

Two forms of data were collected in this study: (1) scores obtained by the students to appraise their academic achievement, and (2) data from a survey on teacher satisfaction with the program. Therefore, the following instruments were used:

(1) Academic performance according to student school records. Scores were assessed in seven areas: Natural Science, Social Science, Spanish Language and Literature, Mathematics, Foreign Language, Regional Language, Physical Education, and an average grade (an average of the scores obtained in each area of knowledge).
(2) Satisfaction survey. The survey was administered with the objective of determining teachers’ degree of satisfaction with the 7-week training program upon its completion. The survey consisted of 10 statements for which subjects rated the extent to which they agreed or disagreed with each on a 5-point Likert scale [1 (“strongly disagree”) to 5 (“strongly agree”)]. The statements were as follows:
  (1) The program has facilitated my *understanding of the use of EI* as a key competency to improve academic primary school achievement.
  (2) The objectives were appropriate for the planned duration and the established *work schedule*.
  (3) I consider the *method* used to be effective.
  (4) I consider the *transfer of knowledge* to my teaching practice understandable.
  (5) I believe the knowledge that I have gained will positively affect my *methods* in the classroom.
  (6) I believe that the competencies that I have gained from the program will help me to better understand my emotions (and the emotions of my students) and to *manage* them successfully.
  (7) The 7-week teacher training program met my *expectations*.
  (8) My *motivation and interest* during the 7-week teacher training program was high.
  (9) I consider this teacher training program a good stimulus for *teacher performance improvement.*
  (10) I consider the training engaging and beneficial for the improvement of *academic achievement in primary school.*

### Procedure

All the teachers were fully informed of the details of the study (including the objectives, the responsible team, the confidentiality of their answers on the survey, and the confidentiality regarding the grades obtained by their students) prior to participation, and their written informed consent was obtained. This study was conducted in accordance with the Declaration of Helsinki.

The participants were randomly assigned to one of two experimental groups or the control group. The first experimental group participated in a 7-week teacher training program designed to improve academic achievement in their primary school students through a face-to-face method. The second experimental group participated in a 7-week teacher training program designed to improve academic achievement in their primary school students through a game-based e-learning method. The control group consisted of primary school teachers who did not participate in the program or receive any other intervention during the 7-week period.

The grades obtained by the students were recorded before and after the 7-week teacher training program in all experimental conditions. In addition, after the 7-week teacher training program, the two experimental groups completed a survey to assess their satisfaction with the program.

### Experimental Design and Data Analysis

To analyze the effect of the 7-week training program on primary school academic achievement, within the general linear model procedure, the univariate split-plot variance was analyzed. Data from the students’ school records were analyzed using repeated-measures analysis of variance (factors: group and time). Tests of within-subjects interaction effects (time × group) were used to identify the effects of the transfer of EI knowledge by the teacher on academic achievement in the students after the 7-week training program. Subsequently, a comparison of means was performed to analyze whether there were significant differences in academic achievement among the experimental (in the two modalities) and control groups. In addition, teacher satisfaction with the 7-week training program was analyzed. Finally, graphs of interactions, illustrating the differences found and their meanings, were created for the experimental groups (in their two modalities) and for the control group in the pre-test and post-test settings. For all statistical analyses, SPSS, version 21.0 (IBM Corporation, Armonk, NY, United States) was used.

## Results

To analyze whether there were differences in academic primary school achievement between the three groups before the intervention, mean contrast for the independent samples was completed. The results showed that there were no significant differences in any of the measured variables between the two groups in the pre-training. In addition, results of a Box’s *M* test did not show homogeneity of the variance-covariance matrix for the Natural Science student records (*M* = 96.25; *F* = 16.02; *p* ≥ 0.00), Social Science student records (*M* = 104.17; *F* = 17.33; *p* ≥ 0.00), Spanish Language and Literature student records (*M* = 119.25; *F* = 19.84; *p* ≥ 0.00), Mathematics student records (*M* = 98.21; *F* = 16.34; *p* ≥ 0.00), Foreign Language student records (*M* = 104.13; *F* = 17.33; *p* ≥ 0.00), Regional Language student records (*M* = 91.56; *F* = 15.23; *p* ≥ 0.00), Physical Education student records (*M* = 75.14; *F* = 12.50; *p* ≥ 0.00), or on the average grade (*M* = 397.67; *F* = 66.18; *p* ≥ 0.00). It should be noted that a violation of this assumption has a minimum effect if the groups are approximately equal in size (Hair et al., 1999).

Next, contrast tests using analysis of variance showed a significant time × group interaction, as seen in Table 2, of intra-subject and inter-subject effects. The resulting grades from the student school records indicate that the effect of the interaction between the time of evaluation (pre-training and post-training) and the implementation of the program was significant (*p* = <0.01) between the face-to-face instruction group and the game-based e-learning instruction group compared with the control group. Differences between the experimental (in the two modalities) and control groups as reflected by changes in the average grade (and in the score in all knowledge areas) after the 7-week training program.

**TABLE 2:** Summary of intra-inter subject univariate analysis of variance.

	**Source**	**Type III**	**df**	***F***	**Sig.**	**η^2^ partial**	**Ob. Power**	***Post hoc***
Natural Science	Intra	634.13	1	668.77	0.00^∗∗^	0.24	1.00	1 < 2; 2 > 3;
	Intra^∗^Entre	120.61	2	63.60	0.00^∗∗^	0.06	1.00	3 < 1
	Error intra	1958.96	2066					
	Inter	213929.36	1	91157.38	0.00^∗∗^	0.99	1.00	
	Condition	58.56	2	12.47	0.00^∗∗^	0.01	0.99	
	Error inter	4848.51	2066					
Social Science	Intra	751.87	1	805.11	0.00^∗∗^	0.28	1.00	1 < 2; 2 > 3;
	Intra^∗^Entre	123.89	2	66.33	0.00^∗∗^	0.06	1.00	3 < 1
	Error Intra	1929.38	2066					
	Inter	215283.29	1	89673.94	0.00^∗∗^	0.97	1.00	
	Condition	91.04	2	18.96	0.00^∗∗^	0.01	1.00	
	Error inter	4959.91	2066					
Spanish Language	Intra	655.79	1	688.20	0.00^∗∗^	0.25	1.00	1 < 2; 2 > 3;
	Intra^∗^Entre	144.41	2	75.77	0.00^∗∗^	0.06	1.00	3 < 1
	Error Intra	1968.69	2066					
	Inter	216462.77	1	87227.84	0.00^∗∗^	0.97	1.00	
	Condition	64.72	2	13.15	0.00^∗∗^	0.01	0.99	
	Error inter	5084.30	2066					
Mathematics	Intra	713.73	1	790.67	0.00^∗∗^	0.27	1.00	1 < 2; 2 > 3;
	Intra^∗^Entre	123.44	2	68.37	0.00^∗∗^	0.06	1.00	3 < 1
	Error intra	1864.98	2066					
	Inter	215187.86	1	90113.56	000.00^∗∗^	0.98	1.00	
	Condition	90.20	2	18.88	0.00^∗∗^	0.01	1.00	
	Error inter	4933.53	2066					
Foreign Language	Intra	670.43	1	743.88	0.00^∗∗^	0.26	1.00	1 < 2; 2 > 3;
	Intra^∗^Entre	160.33	2	88.95	0.00^∗∗^	0.07	1.00	3 < 1
	Error intra	1861.99	2066					
	Inter	215536.29	1	87022.59	000.00^∗∗^	0.97	1.00	
	Condition	64.20	2	32.10	0.00^∗∗^	0.01	0.99	
	Error inter	5117.03	2066					
Regional Language	Intra	691.91	1	750.84	0.00^∗∗^	0.26	1.00	1 < 2; 2 > 3;
	Intra^∗^Entre	134.53	2	67.26	0.00^∗∗^	0.06	1.00	3 < 1
	Error intra	1903.86	2066					
	Inter	214919.85	1	91597.95	0.00^∗∗^	0.98	1.00	
	Condition	120.42	2	25.64	0.00^∗∗^	0.02	1.00	
	Error inter	4860.71	2066					
Physical Education	Intra	759.40	1	872.68	0.00^∗∗^	0.29	1.00	1 < 2; 2 > 3;
	Intra^∗^Entre	1118.59	2	68.14	0.00^∗∗^	0.06	1.00	3 < 1
	Error intra	1797.81	2066					
	Inter	215001.99	1	86121.07	0.00^∗∗^	0.98	1.00	
	Condition	74.46	2	14.91	0.00^∗∗^	0.01	0.99	
	Error inter	5157.78	2066					
Average grade	Intra	695.79	1	3389.17	0.00^∗∗^	0.62	1.00	1 < 2; 2 > 3;
	Intra^∗^Entre	131.09	2	319.28	0.00^∗∗^	0.23	1.00	3 < 1
	Error intra	195.656	2066					
	Inter	214936.00	1	126391.42	0.00^∗∗^	0.98	1.00	
	Condition	79.09	2	23.25	0.00^∗∗^	0.02	1.00	
	Error inter	3513.35	2066					

*^∗^*p* < 0.05, ^∗∗^*p* < 0.01.*

In addition, this improvement is clear in the two experimental groups, but the best results are achieved in the game-based e-learning group. Table 3 presents the pre-training and post-training means of each of the groups.

**TABLE 3:** Marginal means.

	**Group**	***X***	**SD**	***N***
Natural Science (T1)^∗^	1	6,79	1,16	645
	2	6,77	1,27	758
	3	6,89	1,29	666
Social Science (T1)^∗^	1	6,80	1,18	645
	2	6,77	1,30	758
	3	6,83	1,24	666
Spanish Language (T1)^∗^	1	6,84	1,16	645
	2	6,73	1,32	758
	3	6,89	1,33	666
Mathematics (T1)^∗^	1	6,84	1,12	645
	2	6,76	1,29	758
	3	6,83	1,31	666
Foreign Language (T1)^∗^	1	6,81	1,17	645
	2	6,75	1,35	758
	3	6,93	1,31	666
Regional Language (T1)^∗^	1	6,83	1,14	645
	2	6,79	1,35	758
	3	6,82	1,26	666
Physical Education (T1)^∗^	1	6,83	1,16	645
	2	6,73	1,27	758
	3	6,82	1,29	666
Average Grade (T1)^∗^	1	6,82	0,94	645
	2	6,76	0,98	758
	3	6,86	0,96	666
Natural Science (T2)^∗∗^	1	7,67	1,16	645
	2	7,91	1,23	758
	3	7,22	1,54	666
Social Science (T2)^∗∗^	1	7,72	1,18	645
	2	8,01	1,22	758
	3	7,24	1,58	666
Spanish Language (T2)^∗∗^	1	7,70	1,17	645
	2	7,95	1,24	758
	3	7,21	1,56	666
Mathematics (T2)^∗∗^	1	7,69	1,15	645
	2	8,01	1,25	758
	3	7,24	1,53	666
Foreign Language (T2)^∗∗^	1	7,71	1,17	645
	2	7,99	1,23	758
	3	7,22	1,53	666
Regional Language (T2)^∗∗^	1	7,76	1,14	645
	2	7,99	1,24	758
	3	7,16	1,49	666
Physical Education (T2)^∗∗^	1	7,69	1,16	645
	2	8,00	1,30	758
	3	7,27	1,55	666
Average Grade (T2)^∗∗^	1	7,71	0,89	7,71
	2	7,98	0,74	7,98
	3	7,22	1,28	7,22

*^∗^Time 1 (T1)^∗^ = pre-training, ^∗∗^ Time 2 (T2)^∗^ = post-training.*

**FIGURE 1:**
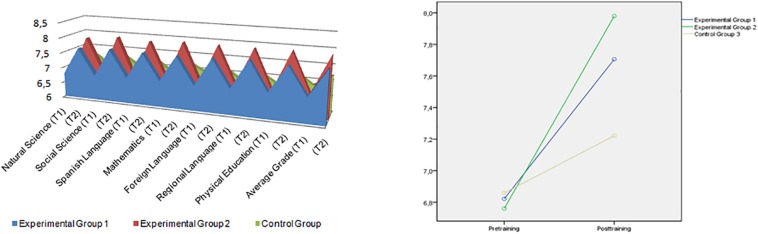
Changes by subject and average grade for group 1 (face-to-face methodology), group 2 (e-learning gamification methodology), and control group with the implementation of a 7-week training program and administration of two tests (before and after the training).

Figure 1 presents interaction graphs that illustrate directions of the differences by subject and, as a conclusion, for average grades. It is important to state that there were just two tests (before and after the training) for each of the subjects and for the average mark. In this way, the results show that after the 7-week training period, significant changes were observed in students’ school records overall for the face-to-face instruction and game-based e-learning instruction groups, compared with the control group, demonstrating the enhancement of coping strategies after completion of the 7-week program.

With regard to teachers’ degree of satisfaction with the 7-week training program, the two experimental groups demonstrated high scores for all the statements. Ninety-seven percent of the teachers in the two groups strongly agreed with all the survey items. However, the game-based e-learning instruction group demonstrated significantly high scores for statement 3 (“I consider the method used to be effective”) and statement 5 (“I believe the knowledge that I have gained will positively affect my methods in the classroom.”). In that group, 100% of teachers strongly agreed with both items.

## Discussion

In recent decades, there has been growing evidence of the importance of EI competency in primary school (Karavasilis et al., 2010; O’Rourke et al., 2010; Kopecki and Katavic, 2011; O’Connor, 2012; Pangiras et al., 2012; Baumeister et al., 2014; Barmpouti et al., 2015; Ferres et al., 2018). The new competencies needed for academic achievement in primary school are no longer exclusively academic (Aslam, 2018; Howard and Cogswell, 2018; Vahabzadeh et al., 2018; Vasilieva, 2018). Other types of skills such as EI and everything that EI implies (e.g., self-esteem, self-realization, emotional self-consciousness, positive relationships, empathy, and social responsibility) are also factors in academic achievement.

In addition, the EI behavioral approach demonstrates that competencies and talent can be developed across the right training programs in different contexts (education, organizations, etc.) and improvement can be sustained for years (Boyatzis, 2006, 2008). Therefore, it is important in EI training to provide evidence for the relationships between EI competencies and a person’s actions, life outcomes, performance, engagement, citizenship, and innovation beyond general mental ability and personality traits (Boyatzis, 2018).

This study showed that academic performance, measured by student school records, improved after teacher participation in a 7-week teacher training program on the implementation of EI as a key competency in teaching, across two different methods: (1) face-to-face instruction and (2) game-based e-learning instruction. The findings of this study indicate that it is possible to improve academic achievement across the two methods; however, similar to other studies (Mysirlaki and Paraskeva, 2015; Ros-Morente et al., 2018; Gray et al., 2019; Nikolaou et al., 2019), the improvement was found to be more significant when game-based e-learning was used. In addition, teacher satisfaction with the 7-week training program was very high for both methods.

## Conclusion

In conclusion, EI is a key factor for educational achievement. Therefore, teachers must be qualified to teach EI in the classroom. These findings suggest that EI can be taught with different training methods using new pedagogical designs. However, one limitation of the present paper is that no follow-up survey was administered until some time had passed after the training had ended; therefore, the time period for realizing positive results of the training is not known. For this reason, further research on this topic is warranted to improve the quality of teaching in primary school.

## Data Availability Statement

The datasets generated for this study are available on request to the corresponding author.

## Ethics Statement

The studies involving human participants were reviewed and approved by the University of Alicante Ethics Committee (UA-2015-07-06). The ethics committee waived the requirement of written informed consent for participation.

## Author Contributions

TP-R was responsible of the research, carried out the quantitative methods, and did fieldwork and program implementation. IS conducted the theoretical review of the topic.

## Conflict of Interest

The authors declare that the research was conducted in the absence of any commercial or financial relationships that could be construed as a potential conflict of interest.
